# Impact of augmentation strategy variations on the mechanical characteristics of patients with osteoporotic proximal humerus fractures with medial column instability

**DOI:** 10.3389/fbioe.2024.1463047

**Published:** 2024-09-25

**Authors:** Guoqing Xiao, Xiang Zhang, Alin Duan, Jian Li, Jialei Chen

**Affiliations:** ^1^ Sports Medicine Center, Department of Orthopedic Surgery, West China Hospital, Sichuan University, Chengdu, Sichuan, China; ^2^ Department of Orthopedic Surgery, The Second Affiliated Hospital of Chengdu Medical College, China National Nuclear Corporation 416 Hospital, Chengdu, Sichuan, China; ^3^ Department of Orthopedic Surgery, West China Hospital, Sichuan University, Chengdu, Sichuan, China

**Keywords:** proximal humeral fractures, medial support, osteoporosis, biomechanics, finite element analysis

## Abstract

**Introduction:**

Low bone density and lack of medial support are the two most important factors affecting the stability of locking plate fixation for osteoporotic proximal humeral fractures (PHFs). This study aimed to compare the biomechanical characteristics of PHILOS locking plates combined with calcar screws, bone cement, fibular allografts, and medial locking plate support strategies for treating osteoporotic PHFs with medial column instability.

**Methods:**

A three-part osteoporotic PHF (AO 11-B3.2) model with metaphyseal loss was generated using 40 synthetic humeri and fixed via four distinct medial support strategies. All models were mechanically tested to quantify the mechanical characteristics. Subsequently, finite element models were created for each biomechanical test case. The stress distribution and displacement of the four different fixation structures were analyzed using finite element analysis.

**Results:**

The results demonstrated that the PHILOS locking plate combined with the medial locking plate, exhibited the greatest stability when subjected to axial, shear, and torsional loading. Furthermore, the PHILOS locking plate combined with bone cement showed structural stability similar to that of the PHILOS locking plate combined with fibular allograft but with lower stress levels on the fracture surface.

**Discussion:**

In conclusion, the PLP-MLP fixation structure showed superior biomechanical properties under axial, shear, and torsional loading compared to other medial support methods. Repairing the medial support when treating osteoporotic PHFs with medial column instability can enhance the mechanical stability of the fracture end in both the short and long term.

## 1 Introduction

As the second most common upper limb fracture, proximal humerus fractures (PHFs) are commonly found in individuals over the age of 65 years who have osteoporosis and are associated with a high mortality rate (Koeppe et al., 2023; Sumrein et al., 2023). Severe fractures in patients are caused by a combination of advanced age, osteoporosis, and poor initial displacement (Foruria et al., 2011). There is clear evidence that restoring the anatomy of the proximal humerus and maintaining the stability of the fracture ends are particularly crucial for improving the long-term prognosis of the shoulder joint in such patients (Miltenberg et al., 2022). Compared with standard nonlocking plates, locking plates are the preferred therapy for osteoporotic and comminuted PHFs due to the advantages of higher failure loads, less damage to soft tissues, and the ability to provide multidirectional fixation (Röderer et al., 2011). Subsequent studies conducted over a long period have demonstrated that most patients with displaced and unstable PHFs who undergo treatment with locking plates experience positive results (Ockert et al., 2014). Nevertheless, despite the biomechanical benefits, the occurrence of screw penetration and varus deformity after using locking plate fixation for PHF is as high as 44%. This complication is more prevalent in patients with osteoporotic PHF who also have medial column loss or epiphyseal comminution (Kralinger et al., 2014; Barlow et al., 2020). Osteoporotic PHFs are characterized by a cancellous bone deficiency in the central part of the humeral head (Carbone et al., 2018). The screws need to be long enough to reach the subchondral bone, increasing the risk of screw penetration (Erhardt et al., 2012). Furthermore, comminution of the medial column of the humeral neck due to reduced bone mass further reduces the mechanical stability of implant fixation.

The metaphyseal bone defect caused by fracture comminution is the underlying cause of the elevated risk of postoperative complications in patients with osteoporotic PHFs (Zeng et al., 2018). Both clinical practice and biomechanical studies have demonstrated that medial support can be augmented by using a combination of calcar screws, autogenous bone grafts, allograft bone grafts, bone cement, and dual-plate fixation methods to enhance the stability of the fracture ends (Sun et al., 2020). However, no study has comprehensively analyzed the biomechanical characteristics of these different medial support methods (Zhang et al., 2014; Yang et al., 2015). This study aimed to compare the biomechanical stability of different medial support augmentation strategies for treating osteoporotic PHFs with medial column instability through biomechanical tests and finite element analysis (FEA), providing a biomechanical basis for selecting suitable medial support augmentation methods in clinical practice.

## 2 Materials and methods

### 2.1 Fracture model preparation

Forty synthetic humeri (LSH5350, Synbone, Sweden) of the same size and density were used for biomechanical studies. The distal section of all humerus models was partially resected, and the proximal 20 cm length was retained. A 5 cm section of the distal humerus was then encapsulated by embedding it in a square of polymethylmethacrylate to provide a secure fixation of the distal humerus (Cristofolini et al., 2021). Previous studies have shown that synthetic bone can represent the anatomical morphology of the humerus in most individuals and is a suitable alternative to using cadaveric bone for biomechanical studies (Lescheid et al., 2010; Grover et al., 2011). A three-part osteoporotic PHF with a metaphyseal loss model (AO 11-B3.2) was simulated using osteotomy techniques based on the osteotomy protocol developed by Tilton et al. (2020).

First, an osteotomy was performed below the humeral diaphysis, parallel to the surgical neck. Then, a second osteotomy was performed 10 mm below the first osteotomy to mimic a comminuted metaphysis fracture. The third cut was a vertical osteotomy along the greater tuberosity-intertrochanteric groove to detach the greater tuberosity from the humeral head and shaft (Supplementary Figure 1). Furthermore, to simulate the loss of medial support under severe osteoporotic conditions [21], a ø30 mm drill bit was employed to extract the internal cancellous bone of the humeral head in each synthetic specimen while retaining 40% of the cancellous bone volume to imitate an “eggshell defect” in the humeral head (Feerick et al., 2013). This fracture type represents a severe injury lacking medial cortical support and is predominant in elderly osteoporotic patients (Handoll et al., 2022).

### 2.2 Surgical techniques and grouping

**FIGURE 1:**
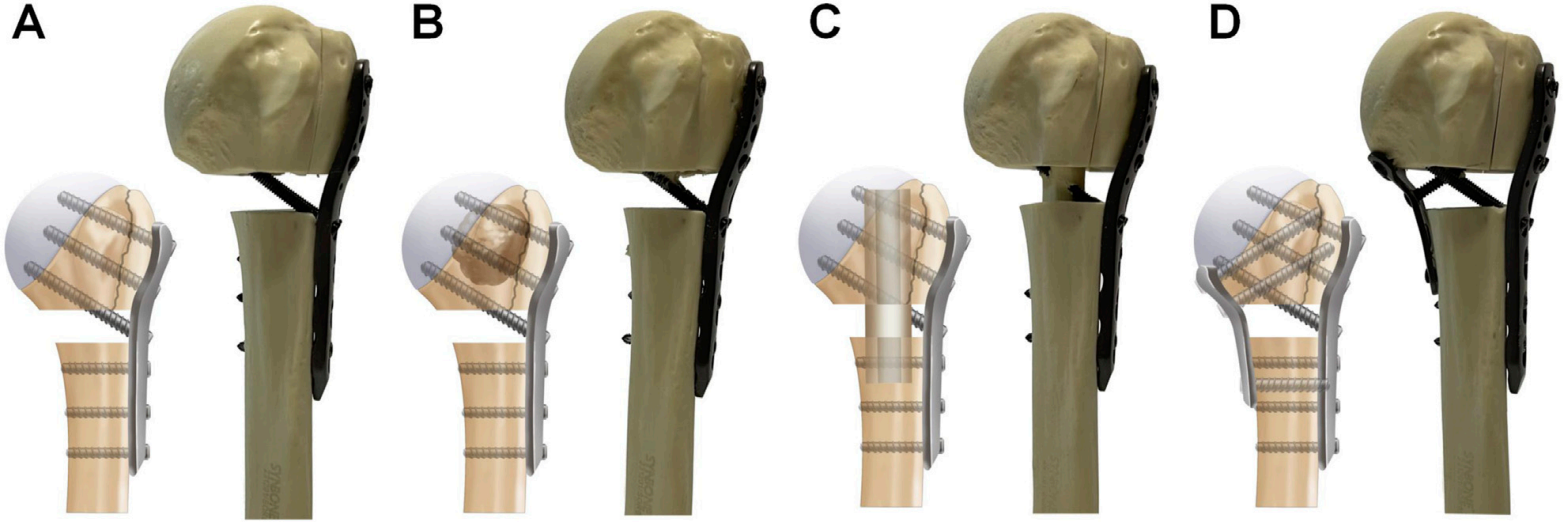
Schematic representation of the four medial support methods for fixation. **(A)** PLP-CS, PHILOS locking plate combined with the medial support of calcar screws; **(B)** PLP-BC, PHILOS locking plate combined with bone cement augmentation; **(C)** PLP-FA, PHILOS locking plate combined with the medial support of a fibular allograft; **(D)** PLP-MLP, PHILOS locking plate combined with a medial locking plate.

An experienced orthopedic surgeon reconstructed all of the PHF models. Identical PHILOS locking plates (JIASKANG, China) were used in all fracture models. The plates were placed 1 cm below the greater tuberosity following the recommended guidelines (Omid et al., 2021; Zhelev et al., 2023). Locking screws of appropriate lengths (6 proximal and 3 distal) were chosen based on the measurement of the probing depth. All fracture models were randomly divided into 4 groups of 10 specimens each: 1. PHILOS locking plate combined with medial support of calcar screws (PLP-CS, Figure 1A). The PHF was stabilized using a PHILOS locking plate, and 6 locking screws were placed into the humeral head fragment, with all of the proximal screws at a distance of more than 5–8 mm from the subchondral bone to avoid screw penetration [24]. Three locking screws were used to fix the humeral shaft fragment. 2. PHILOS locking plate combined with bone cement augmentation (PLP-BC, Figure 1B). Based on the PLP-CS fixation construct, 8 mL of medium-viscosity bone cement (PALACOS^®^, Germany) was manually placed through the lateral window of the fracture into the humeral head fracture fragment to fill the humeral head (Zhelev et al., 2023). 3. PHILOS locking plate combined with the medial support of a fibular allograft (PLP-FA, Figure 1C). Based on the PLP-CS fixation structure, a 60 mm allograft fibula was implanted into the fracture model and secured using locking screws, with the upper part of the fibula fixed below the cortical apex of the humeral head. The allograft fibula was positioned close to the medial cortical bone to improve the medial support (Cui et al., 2019). 4. PHILOS locking plate combined with a medial locking plate (PLP-MLP, Figure 1D). Based on the PLP-CS fixation structure, the medial side was fixed with a three-hole locking plate (JIASKANG, China) to provide medial support.

### 2.3 Biomechanical tests

**FIGURE 2:**
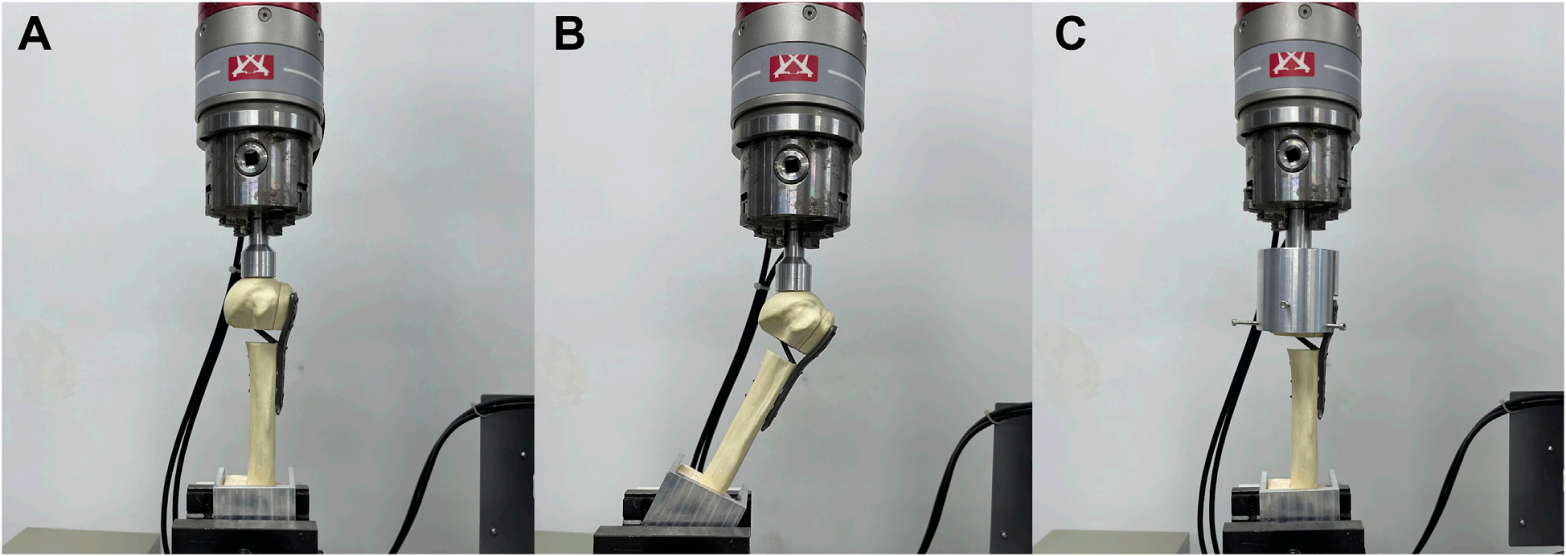
Three types of loading performed in biomechanical tests. The distal fragment of the humeral shaft was embedded in square polymethylmethacrylate for encapsulation. **(A)** Axial force, **(B)** shear force and **(C)** torsional forces were applied to the models.

The fracture models were fixed using a customized XY table and subjected to biomechanical tests on an INSTRON E3000 series universal mechanical testing machine (INSTRON Corporation, United States). Axial, shear, and torsional loads were applied to each group of fixed models to test their structural stiffness (Figure 2) (Zhang et al., 2014). For axial stiffness, a vertical load (preload = 50 N) was applied to the tip of the humeral head at a rate of 5 mm/min until the humeral head fragment was displaced vertically up to 0.5 mm. In the shear loading test, the model’s angle was adjusted by 20° following the axial condition to simulate the shear force on the fractured end when the patient was standing with abductor weight, except that the maximum displacement was set at 1 mm. To test the torsional stiffness, a displacement controller was used to apply torque at a rate of 12°/min (maximum angle = 5°, pretorque = 0 N m) to simulate the rotating movement of the humeral head in the glenoid. The maximum load and the torque were recorded for each group, and the structural stiffness was determined by fitting the slope. All fracture fixation model deformations were within the elastic range of the line to prevent bone and fixation structure damage.

All models that underwent stiffness tests were subjected to cyclic shear loading tests to assess the long-term stability of the fracture ends fixed with different medial support methods during postoperative shoulder functional exercises. According to previous studies, a set of 1,000 cycles was programmed into the software Instron Wave Matrix2 (INSTRON Corporation, United States), and cyclic shear loads varying from 50 N to 623 N were applied to the humeral head at 1 Hz (Burke et al., 2014). Cycle‒displacement curves were recorded. At the end of the cyclic shear test, a shear load (preload = 50 N) was applied to each fracture model at a rate of 5 mm/min for the destructive experiment until fixation failure occurred. Fixation failure was defined as plate or screw bending, screw cutting, the appearance of new fracture lines, a relative displacement of the fracture end greater than 5 mm (Neer et al., 1970) considered ≥5 mm displacement to be an indication for surgical treatment of PHFs, and a sudden change in the load-displacement curve.

### 2.4 Finite element modelling

The overall FEA workflow is illustrated in Supplementary Figure 2. CT images of the synthetic humerus (LSH5350, Synbone, Sweden) were imported into Mimics 21.0 (The Materialise Group, Belgium) for 3D modelling of the proximal humerus. The 3D model of the proximal humerus in the STL format was imported into Geomagic Wrap 2021 (Geomagic, United States) for further surface processing. Subsequently, Boolean operations were employed in SolidWorks 2021 (SolidWorks, United States) to segment the proximal humerus cortical and cancellous bone models. A three-part osteoporotic PHF with medial column deficiency (AO 11-B3.2) was constructed according to the osteotomy protocol used in the biomechanical experiments. Three-dimensional models of the PHILOS locking plate, locking screws, bone cement, fibular allograft, and three-hole locking plate were constructed in SolidWorks 2021 software according to the dimensional information provided by the manufacturer. The internal fixation models were assembled and grouped with the fracture models based on the biomechanical experimental fixation scheme.

Meshing was performed using a tetrahedral ten-node cell (C3D10) with a size of 1 mm based on the mesh planning element size in previous study (Yang et al., 2015). The numbers of nodes and elements for each group of models are shown in Table 1. Subsequently, four distinct medial support augmentation models were imported into ANSYS Workbench 2020 R2 (Ansys, Canonsburg, PA) for FEA. All models were assumed to be homogeneous, isotropic linear elastic materials. Young’s modulus and Poisson’s ratio of each model are shown in Table 2 (Kennedy et al., 2013a; Yang et al., 2015; Chen et al., 2020). Friction contact was defined as friction between the fracture ends and the plate-bone interface, with friction coefficients of 0.46 and 0.3, respectively. The interfaces between the screw-plate, screw-bone, and screw-cement interfaces were defined as bounded contacts.

**TABLE 1:** Element information consisting of finite element models.

Finite element models	PLP-CS	PLP-BC	PLP-FA	PLP-MLP
Number of nodes	390,793	406,581	396,361	396,964
Number of elements	254,486	264,987	257,087	256,337
Size of element, mm				
Mean	0.79	0.78	0.79	0.78
Maximum	1.00	1.00	1.00	0.99
Minimum	1.55 × 10^−2^	1.54 × 10^−2^	1.54 × 10^−2^	0.92 × 10^−2^

**TABLE 2:** Material properties of models in finite element analysis.

Material types	Young’s modulus, MPa	Poisson’s ratio
Osteoporotic cortical bone	8,844	0.3
Osteoporotic cancellous bone	660	0.3
Titanium alloy (Ti-6AL-7Nb)	13,400	0.3
Fibular allograft	1,520	0.3
Bone cement	110,000	0.3

### 2.5 Boundary conditions and load settings

**FIGURE 3:**
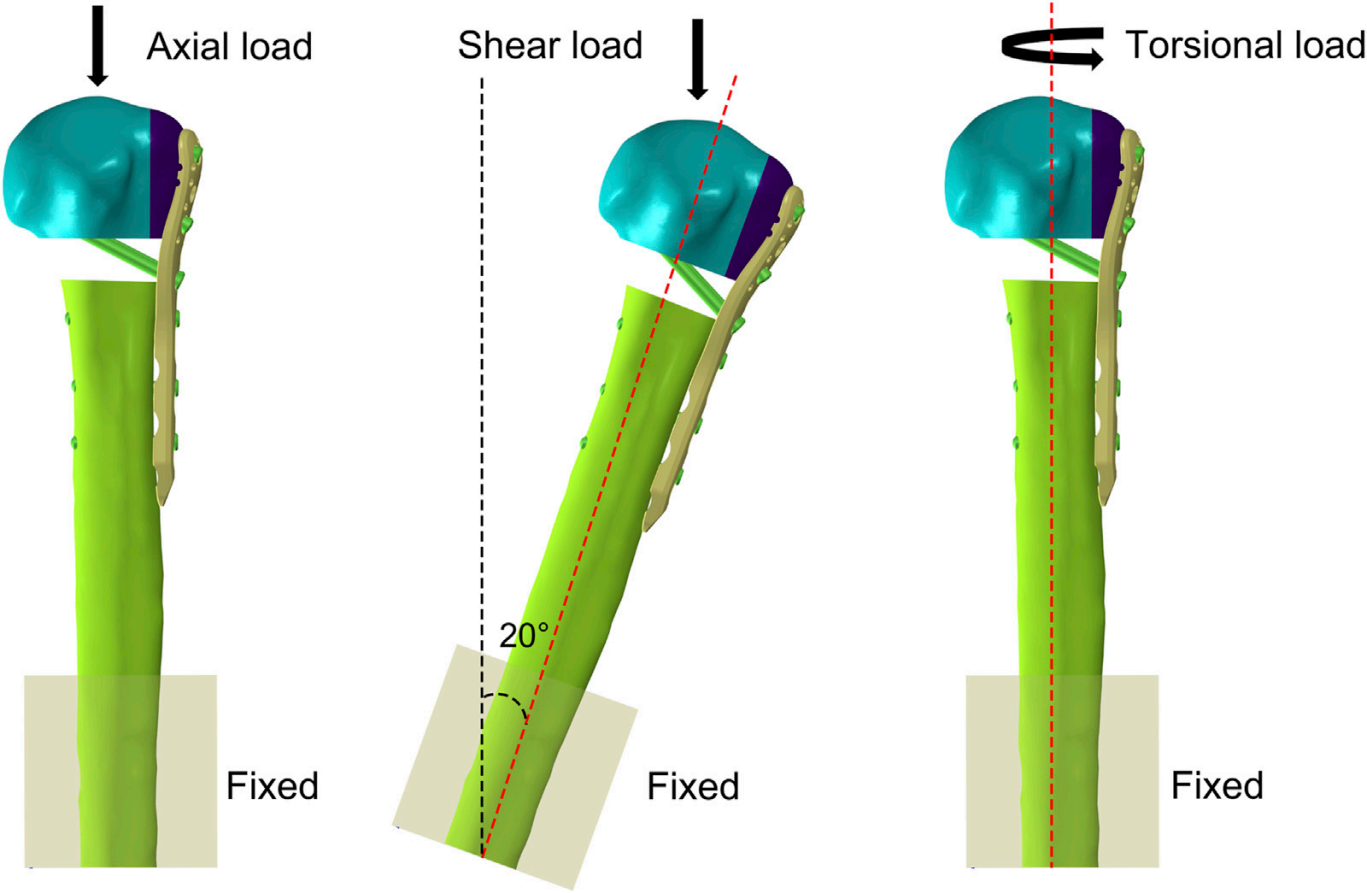
Boundary condition settings in FEA. The freedom of the distal humerus was restricted to 0. Axial, shear, and torsional load were applied to the models.

**FIGURE 4:**
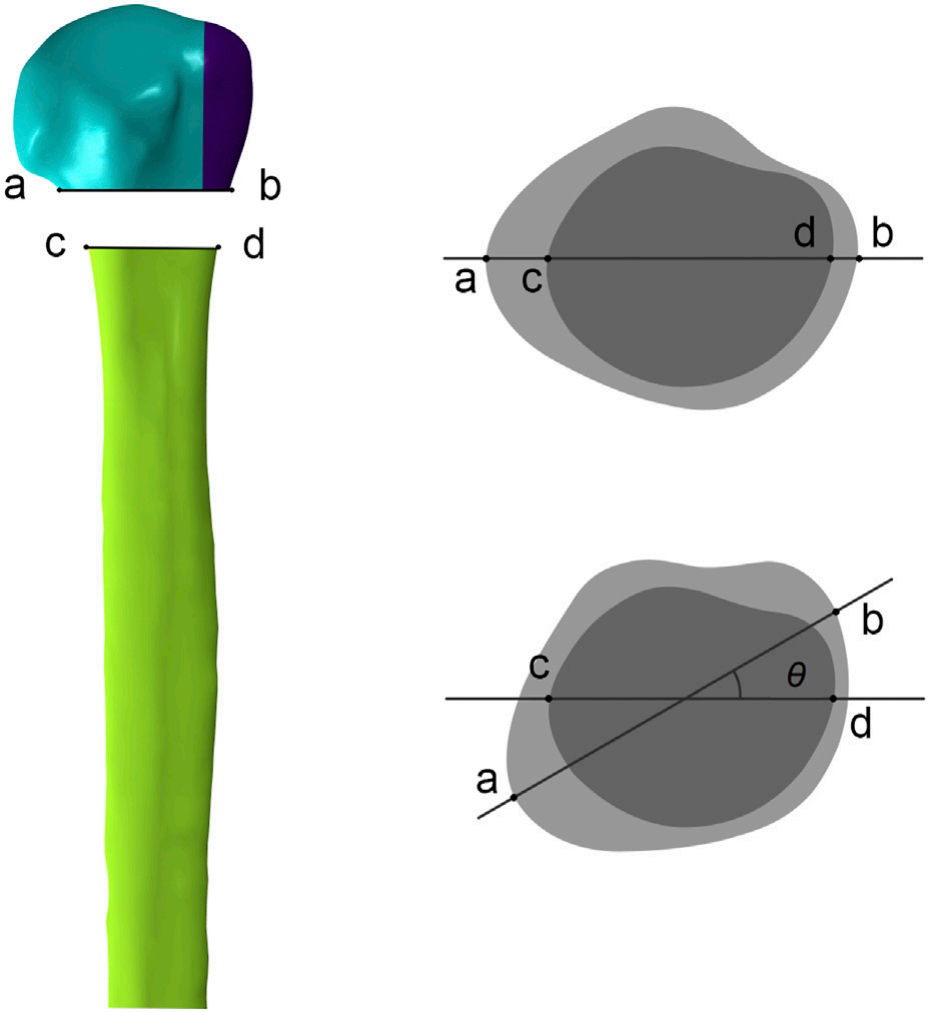
Measurement of the torsional angle (*θ*) of the humeral head relative to the humeral shaft after torsional loading tests. In anterior-posterior view, **ab** represents the line connecting the fractured ends of the humeral head on the front view, and **cd** represents the line connecting the fractured ends of the humeral shaft. In top view, the torsional angle (*θ*) is obtained by measuring the angle between **ab** and **cd** after applying the torsional load.

The freedom of the distal humerus was restricted to 0. Axial, shear, and rotational load applications were applied according to the biomechanical experimental protocol (Figure 3). To simulate axial loads, a load of 500 N was applied vertically in the coronal and sagittal planes (He et al., 2017). The model was made to abduct by 20° to simulate the shear force on the proximal humerus when the patient was standing up from a chair or weight-bearing on crutches. A torque of 3.5 N m was applied around the humeral shaft to simulate torsional loading (Chen et al., 2020). We recorded and analyzed the maximum humeral head-shaft relative displacement (HSRD), maximum torsional angle (TA) (Figure 4), maximum implant Von Misses stress (IVMS), maximum humeral head-greater tuberosity fracture surface Von Misses stress (HGFVMS), maximum head-greater tuberosity fracture surface strain (HGFS), and internal fixation stiffness [stiffness (N/mm) = load (N)/displacement (mm)] under different loading conditions to assess the biomechanical stability of PHF fixation augmented by different medial support methods.

### 2.6 Statistical analysis

Statistical analysis was performed with GraphPad Prism 9. The Shapiro-Wilk test was used to test the normality of the experimental data. If the data of each group conformed to a normal distribution, the ANOVA was used to compare the groups, and the LSD-test was used for two-way comparisons between groups; if not, the Kruskal-wallis test was used to compare the groups, and Bonferroni’s correction was used for two-way comparisons between groups. The level of significance was set to 0.05.

## 3 Results

### 3.1 Stiffness

**FIGURE 5:**
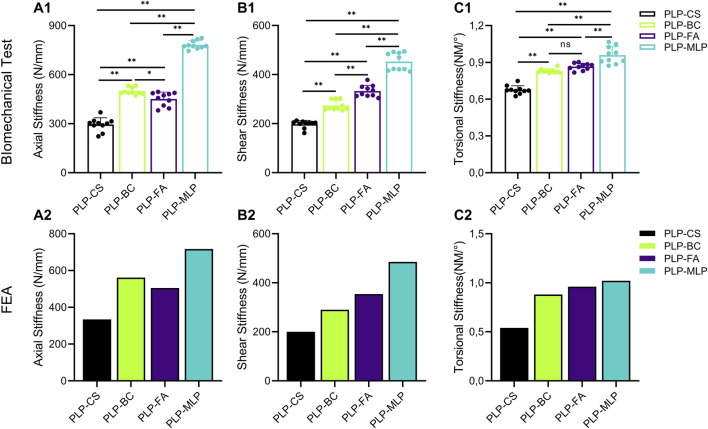
The Structural stiffness of different medial supporting methods measured by biomechanical tests and FEA. **(A1,A2)** axial stiffness; **(B1,B2)** shear stiffness; **(C1,C2)** torsional stiffness (*p < 0.05).

The stiffness values obtained from the biomechanical tests and FEA were within ±1 standard deviation, validating the reliability of the finite element modelling and demonstrating that the modelling approach is suitable for further research. Biomechanical experiments (Figures 5A1–C1; Supplementary Table 1). The axial, shear, and torsional stiffnesses of the PLP-CS group were the smallest among all of the groups, at 295 N/mm ± 41 N/mm, 198 N/mm ± 15 N/mm, and 0.68 N m/° ± 0.03 N m/°, respectively, while those of the PLP-MLP group were the largest, which were 2.7, 2.3 and 1.4 times greater than those of the PLP-CS group (*p* < 0.05). The axial stiffness of the PLP-BC group was greater than that of the PLP-FA group (*p* < 0.05), while the shear stiffness results were the opposite (*p* < 0.05), and the torsional stiffness was close to that of both groups (*p* > 0.05). The FEA results showed the same trend (Figures 5A2–C2; Supplementary Table 1). The axial, shear, and torsional stiffnesses of the PLP-CS group were the smallest among all of the groups, with values of 334 N/mm, 200 N/mm, and 0.54 N m/°, respectively. In contrast, the PLP-MLP group had the highest stiffness. The axial stiffness of the PLP-BC group (562 N/mm) was greater than that of the PLP-FA group (505 N/mm), with opposite results for shear and torsional stiffness.

### 3.2 Cyclic loading test

**FIGURE 6:**
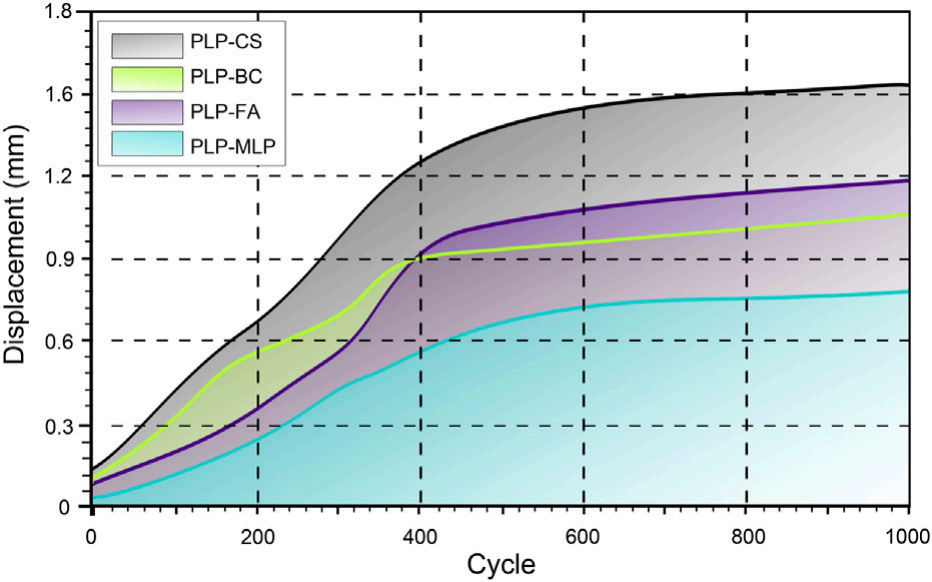
Humeral head and shaft relative displacement analysis of the humeral head and shaft during cyclic tests. The shaded region indicates the range of the displacement for each group. The X-axis starts from 20 cycles.

After 1,000 cycles of cyclic shear loading, the HSRD in the PLP-CS group (1.49 ± 0.17 mm) was approximately twice as high as that in the PLP-MLP group; the HSRD in the PLP-FA group (1.16 ± 0.19 mm) was greater than that in the PLP-BC group (1.03 ± 0.07). Interestingly, the HSRD was lower in the PLP-FA group than in the PLP-BC group before the 400th loading cycle, whereas the opposite result was shown after the 400th cycle (Figure 6; Supplementary Table 2).

### 3.3 Destructive test

**FIGURE 7:**
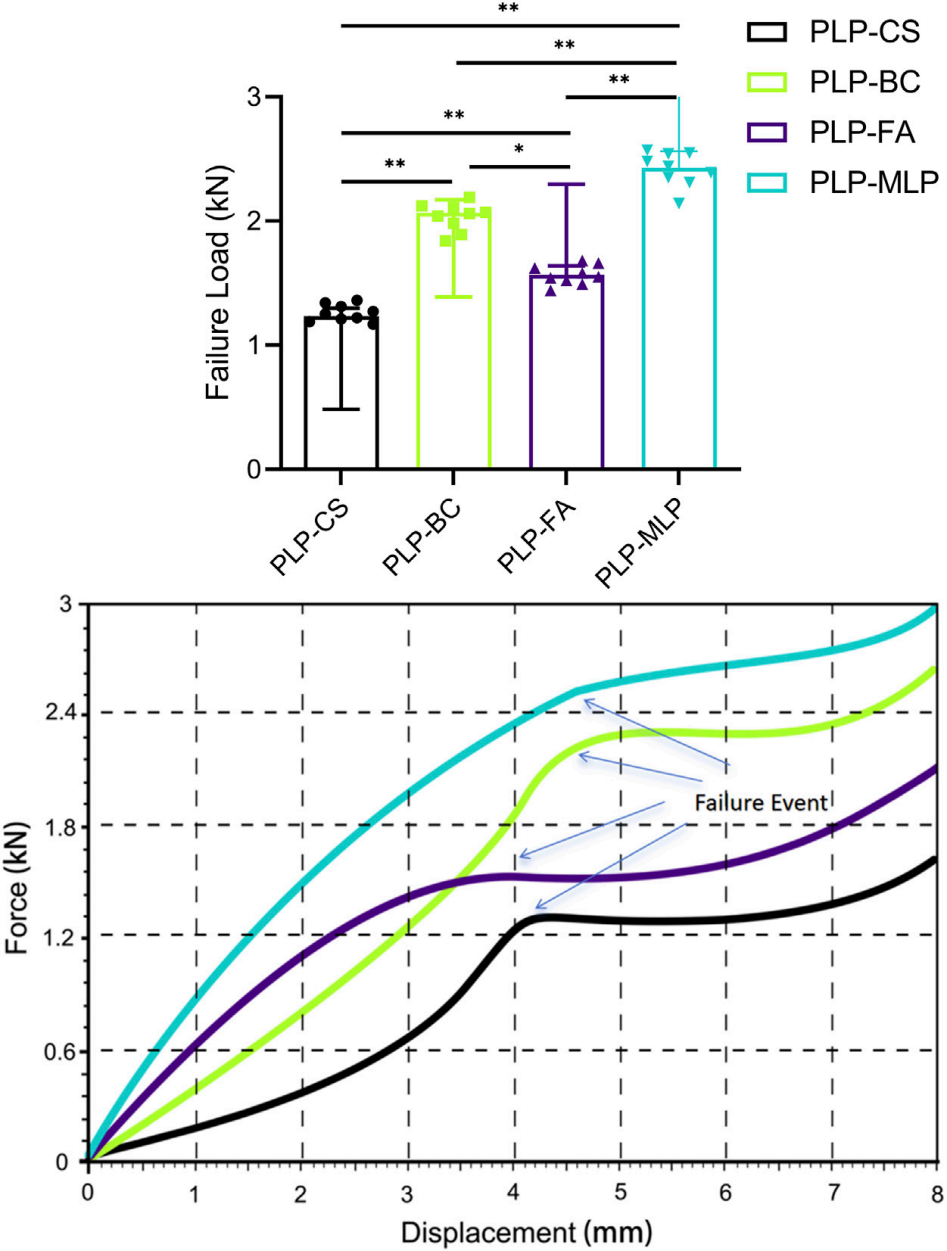
Typical displacement-force diagrams for each group throughout the mechanical destructive test. Although the failure criterion was a migration of 5 mm, the test was extended as far as 8 mm to ensure that failure became evident in all specimens (*p < 0.05).

The results of the destructive test (Figure 7; Supplementary Table 3) showed that the PLP-MLP fixation structure exhibited the highest failure load (2.43 kN ± 0.14 kN), approximately twice as high as that of the PLP-CS fixation structure. The PLP-BC fixation structure (2.04 kN ± 0.11 kN) had a greater failure load than did the PLP-FA (1.57 kN ± 0.07 kN).

### 3.4 FEA

**FIGURE 8:**
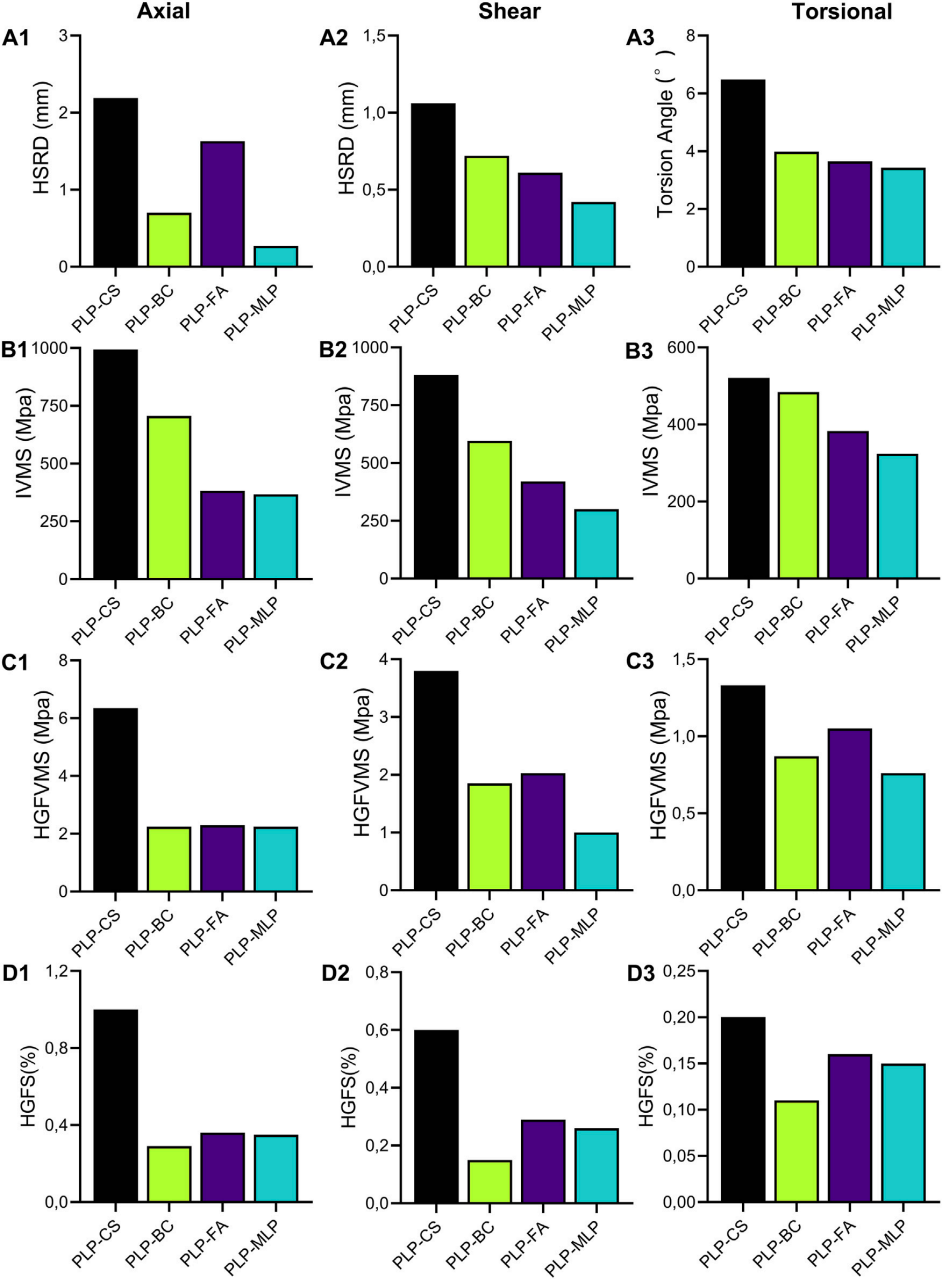
The maximum humeral head-shaft relative displacement (HSRD) **(A1,A2)**, maximum torsional angle **(A3)**, maximum implant Von Misses stress (IVMS) **(B1–B3)**, maximum humeral head-greater tuberosity fracture surface Von Misses stress (HGFVMS) **(C1–C3)**, and maximum head-greater tuberosity fracture surface strain (HGFS) **(D1–D3)** for each group under axial, shear, and torsional loading.

Under axial and shear loading, the HSRD was significantly greater for the PLP-CS group (2.19 mm, 1.06 mm) than for the PLP-MLP group (0.27 mm, 0.42 mm). Although the HSRD of the PLP-FA group was greater than that of the PLP-BC group under axial loading, the opposite results were obtained under shear loading (Figures 8A1, A2; Supplementary Table 4). Under a torque of 3.5 Nm, the torsion angle of the PLP-CS group was the largest at 6.68°, and the TAs of the PLP-BC, PLP-FA, and PLP-MLP fixed structures were similar (Figure 8A3; Supplementary Table 4).

**FIGURE 9:**
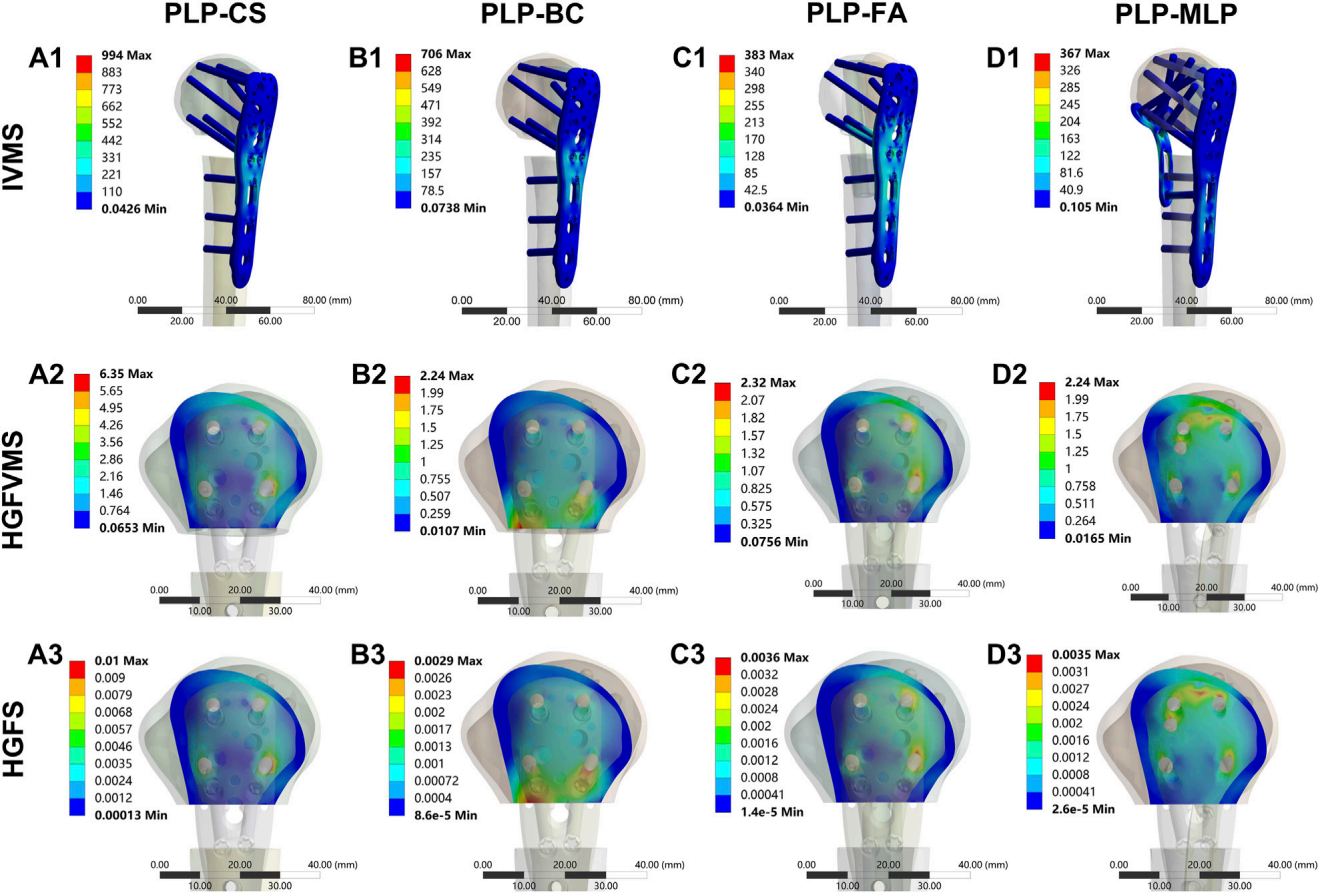
Distribution of IVMS **(A1–D1)**, HGFVMS **(A2–D2)**, and HGFS **(A3–D3)** for PHFs fixed by different medial support methods under axial loading.

The nephograms and IVMS results are shown in (Figures 8B1–B3, Figures 9A1–D1, Supplementary Figures 3, 4). The IVMS of the PLP-CS group under axial, shear, and torsional loads was the largest among all of the groups, at 994 MPa, 881 MPa, and 521 MPa, respectively; that of the PLP-MLP group was the smallest; and that of the PLP-BC group was larger than that of the PLP-FA group. According to the VMS distribution nephograms, the maximum VMS in the PLP-CS and PLP-BC groups was mainly concentrated at the locking plate in the bone defect region, suggesting a greater risk of failure. In contrast, the stress distribution in the PLP-FA group tended to be relatively dispersed, with the fibular allografts sharing part of the stress. The IVMS of the PHILOS locking plate in the PLP-MLP group was significantly lower than that in the other groups, with the medial locking plate accepting most of the stress. The HGFVMS results showed a similar trend (Figures 8C1–C3; Figures 9A2–D2). The HGFVMS under axial, shear, and torsional loads were the largest in the PLP-CS group and the smallest in the PLP-MLP group. In addition, the HGFVMS in the PLP-BC group was lower than that in the PLP-FA group. The PLP-BC group exhibited the smallest HGFS under different loads, and the rest of the groups had increased HGFS, which was mainly concentrated at the bone-screw interface (Figures 8D1–D3; Figures 9A3–D3).

## 4 Discussion

Fractures in elderly osteoporotic patients are usually comminuted, and the medial column fragments of the proximal humerus are prone to be missed after fracture reduction is achieved (Klahs et al., 2024). Due to the lack of medial support, the postoperative complication rates of fixation failure, bone nonunion, malunion, and humeral head necrosis remain high in these patients (Laux et al., 2017). Choosing an appropriate medial support method to reconstruct the medial column can effectively reduce the risk of postoperative complications and improve the long-term prognosis of these patients. This study systematically investigated the biomechanical characteristics of PLP-CS, PLP-BC, PLP-FA, and PLP-MLP fixation structures in treating osteoporotic PHFs with medial column instability. We found that although the PLP-CS fixation structure stabilized and fixed the PHF under axial, shear, and torsional loading, the stability of fracture end fixation was significantly enhanced with the introduction of bone cement, fibular allografts, and medial locking plates, reducing the risk of fixation failure.

The biomechanical stability of the fracture end is a crucial determinant of the healing process for PHF, and deterioration of the biomechanical environment will lead to malunion of the proximal humerus or even nonunion of the fracture (Wright et al., 2021). Although the use of inferomedial calcar screws increases the axial and shear stiffness of PHF fixation, the overall biomechanical stability is not improved (Bai et al., 2014). Therefore, direct medial support may be a more effective strategy. The medial locking plate in the PLP-MLP fixation structure provides direct medial support, and its axial, shear, and torsional stiffnesses are the highest among all of the fixation structures, which agrees with the results of a previous study (Chen et al., 2020). In a retrospective study, Seok and Park (2023) reported that dual-plate fixation for osteoporotic PHF patients with medial column instability and varus deformity had superior imaging and clinical outcomes to single plate fixation. However, introducing medial locking plates in clinical practice may increase the risk of iatrogenic neurovascular injury and subsequent humeral head necrosis. In addition, the PLP-BC fixation structure had greater axial stiffness than the PLP-FA, while the shear stiffness showed the opposite result. In osteoporotic patients, the screw-bone interface is prone to loosening and failure due to reduced bone mineral density (Choma et al., 2011). Bone cement augmentation increased the screw–bone contact area in the area of the bone defect, resulting in a more uniform screw stress distribution and greater resistance to extraction. Furthermore, the bone cement contributed to the bonding of the humeral head-greater tuberosity fracture surface, hence improving its axial rigidity. In contrast, the PLP-FA fixation structure provided direct support and increased the screw-bone contact area in the humeral head fragment. The shear stiffness of the PLP-FA was greater than that of the PLP-BC fixation structure.

Reinforcing the medial support of the proximal humerus can effectively increase the mechanical properties of locking plate fixation for PHFs (Jabran et al., 2018). The cyclic shear loading test results showed that the HSRD significantly decreased after the introduction of different medial support methods, and this change became more pronounced with increasing loading cycles. The medial support structure can share a portion of the load transmitted through the upper and lower sections of the fracture for the PHILOS locking plate, thus reducing the stress concentration in the locking plate and reducing the risk of fixation failure. He et al. (2017) compared the biomechanical properties of four different fixation modalities for the treatment of PHF with medial column instability using FEA and revealed that direct medial support is an effective method for the treatment of PHF with medial column instability. In addition, this study revealed that the use of bone cement increased the overall stiffness of PHF fixation for medial column instability to achieve damage resistance comparable to that of fibular allograft implantation. Interestingly, this study also revealed that PLP-BC fixation was structurally less stable than PLP-FA before 400 loading cycles. However, during the subsequent cyclic cycles, the results were reversed. We hypothesized that during the start of loading period, PLP-FA exhibited greater shear stability than did PLP-BC because of the direct support provided by the fibular allograft. With cyclic loading, the stability of the screw support on the humeral head-greater tuberosity fracture surface decreased in the PLP-FA fixation structure. However, the PLP-BC structure significantly improved the durability of the fixation structure due to a more even distribution of the load per locking screw by the bone cement, which was more pronounced in conditions of osteoporosis-induced bone loss (Kuang et al., 2018). Nevertheless, the risk of cement leakage and the thermal apoptotic necrosis of chondrocytes caused by the exothermic reaction should also be considered when using bone cement (Blazejak et al., 2013).

Destruction experiments effectively measure the secondary stability of different medial bracing schemes (Brunner et al., 2012). The results of the destruction experiment showed that the PLP-MLP fixation structure increased the stability of the head–neck fracture ends due to the support of the medial locking plate, and the humeral head-greater tuberosity fracture surfaces were relatively displaced due to the reduction in the screw–bone contact area and stability, leading to fixation failure. In osteoporotic patients, the central cavity of the humerus lacks cancellous bone, and bone cement matches the mechanical properties of cancellous bone and is an effective filling material (Kennedy et al., 2013b). These findings suggest that from a biomechanical point of view, the PLP-BC fixation structure enhances medial column stability and reduces the risk of fracture end displacement, contributing to improved functional outcomes and a reduced risk of reoperation when treating PHFs with medial column instability. In addition, compared with those of the PLP-BC fixation structure, the FE and biomechanical models of PLP-FA and PLP-MLP are more difficult to construct, implying that these two fixation structures may be more challenging in clinical practice. The FEA stress results further explain the mechanical mechanisms of the biomechanical tests. The stress distribution in the nephograms showed that the stresses in the PLP-MLP and PLP-BC fixation structures were mainly concentrated on the locking plate at the bone defect, which was the main site of fixation failure in the destructive tests. In the PLP-FA fixation structure, the fibular allograft shares part of the stresses of the PHILOS locking plate. However, in the PLP-MLP fixation structure, the medial locking plate shares some of the stress, reducing the risk of fixation failure. With the introduction of enhanced medial support methods, adequate short-term fixation stability is given to the fracture end, contributing to the early healing of the fracture (Jang and Kim, 2021). The HGFVMS of the PLP-BC fixation structure is concentrated on the bone surface close to the defective area of the proximal humerus. In contrast, the HGFVMS of the PLP-CS, PLP-FA, and PLP-MLP fixation structures are mainly concentrated at the bone–screw interface, and the humeral head-greater tuberosity fracture surface tended toward greater separation motion. Although moderate compressive stress on the fracture surface contributes to fracture healing, shear and separation motion at the fracture site is detrimental to scab formation and even increases the risk of postoperative complications (Claes and Meyers, 2020). The HGFS results provide further evidence that the bonding effect of bone cement is effective in decreasing the fracture surface strain and decreasing the risk of trabecular disruption due to bone-screw interface displacements.

There are several limitations in this study. First, the model used in the biomechanical test and FEA was a synthetic humerus, which is somewhat different from cadaveric bone in terms of material properties and anatomical structure. Overall, synthetic bone models could substantially reduce costs and improve the accuracy of simulation results. Second, it is important to note that the complexity of PHF is related not only to the fracture itself but also to the influence of soft tissues such as muscles and ligaments. Despite these limitations, the present study on the biomechanical characterization of different medial support methods to enhance the stability of PHFs can provide a mechanistic reference for clinical decision-making.

## 5 Conclusion

In conclusion, when treating osteoporotic PHFs with medial column instability, restoring the medial support helps to increase the short- and long-term stability of the fracture end. The PLP-MLP fixation structure showed superior biomechanical properties under axial, shear, and torsional loading compared to other medial support methods. In addition, the PLP-BC fixation structure provided adequate stability for PHFs with less damage and easier implementation than PLP-FA, which could be an option for trauma surgeons to treat osteoporotic PHFs with medial column instability.

## Data Availability

The original contributions presented in the study are included in the article/Supplementary Material, further inquiries can be directed to the corresponding author.

## Abbreviations

PHFs, proximal humeral fractures; FEA, finite element analysis; PLP-CS, PHILOS locking plate combined with medial support of calcar screws; PLP-BC, PHILOS locking plate combined with bone cement augmentation; PLP-FA, PHILOS locking plate combined with the medial support of a fibular allograft; PLP-MLP, PHILOS locking plate combined with a medial locking plate; HSRD, humeral head-shaft relative displacement; TA, torsional angle; IVMS, implant Von Misses stress; HGFVMS, humeral head-greater tuberosity fracture surface Von Misses stress; HGFS, humeral head-greater tuberosity fracture surface strain.
