# Supplementary material for: Impact of augmentation strategy variations on the mechanical characteristics of patients with osteoporotic proximal humerus fractures with medial column instability
Source: Front Bioeng Biotechnol. 2024 Sep 25;12:1463047. doi: 10.3389/fbioe.2024.1463047 (PMC11461895; doi:10.3389/fbioe.2024.1463047)
Supplement: Supplementary file 1 [file Table1.DOCX]

**Supplementary Tables**

|  |  | **PLP-CS** | **PLP-BC** | **PLP-FA** | **PLP-MLP** |
| --- | --- | --- | --- | --- | --- |
| **Axial Stiffness(N/mm)** | FEA | 334 | 562 | 505 | 717 |
|  | Biomechanical tests | 295±41 | 500±22 | 451±42 | 782±25 |
| **Shear Stiffness(N/mm)** | FEA | 200 | 290 | 354 | 485 |
|  | Biomechanical tests | 198±15 | 274±20 | 333±24 | 453±36 |
| **Torsional stiffness (N·m/°)** | FEA | 0.54 | 0.88 | 0.96 | 1.02 |
|  | Biomechanical tests | 0.68±0.03 | 0.83±0.02 | 0.87±0.03 | 0.96±0.07 |

**Supplementary Table 1.** The structural stiffness of different medial supporting methods measured by biomechanical tests and FEA.

| **Cycle** | **Humeral head and shaft relative displacement（mm）** | | | |
| --- | --- | --- | --- | --- |
|  | **PLP-CS** | **PLP-BC** | **PLP-FA** | **PLP-MLP** |
| 20 | 0.14±0.03 | 0.12±0.04 | 0.11±0.04 | 0.04±0.03 |
| 200 | 0.69±0.08 | 0.54±0.08 | 0.37±0.05 | 0.24±0.05 |
| 400 | 1.26±0.11 | 0.90±0.09 | 0.93±0.06 | 0.55±0.06 |
| 600 | 1.54±0.13 | 1.06±0.06 | 0.96±0.12 | 0.74±0.06 |
| 800 | 1.61±0.15 | 1.13±0.06 | 1.06±0.16 | 0.78±0.07 |
| 1000 | 1.66±0.17 | 1.18±0.07 | 1.18±0.19 | 0.75±0.11 |

**Supplementary Table 2.** Relative displacement analysis of the humeral head and shaft during cyclic tests.

|  | PLP-CS | PLP-BC | PLP-FA | PLP-MLP |
| --- | --- | --- | --- | --- |
| Failure Load (kN) | 1.25±0.06 | 2.04±0.11 | 1.57±0.07 | 2.43±0.14 |

**Supplementary Table 3.** Maximum destructive load for each group under shear loading.

| **Parameters** |  | | **PLP-CS** | **PLP-BC** | **PLP-FA** | **PLP-MLP** |
| --- | --- | --- | --- | --- | --- | --- |
| HSRD (mm) | | Axial load | 2.19 | 0.7 | 1.63 | 0.27 |
|  |  | Shear load | 1.06 | 0.72 | 0.61 | 0.42 |
| TA (˚) | | Torsional load | 6.48 | 3.98 | 3.65 | 3.43 |
| IVMS (Mpa) | | Axial load | 994 | 706 | 383 | 367 |
|  |  | Shear load | 881 | 596 | 420 | 300 |
|  |  | Torsional load | 521 | 484 | 383 | 324 |
| HGFVMS (Mpa) | | Axial load | 6.35 | 2.24 | 2.3 | 2.24 |
|  |  | Shear load | 3.80 | 1.85 | 2.03 | 1.00 |
|  |  | Torsional load | 1.33 | 0.87 | 1.05 | 0.76 |

**Supplementary Table 4.** The maximum humeral head-shaft relative displacement (HSRD), maximum torsional angle (TA), maximum implant Von Misses stress (IVMS), maximum humeral head-greater tuberosity fracture surface Von Misses stress (HGFVMS) for each group under axial, shear, and torsional loading.
