# Supplementary material for: Impact of augmentation strategy variations on the mechanical characteristics of patients with osteoporotic proximal humerus fractures with medial column instability
Source: Front Bioeng Biotechnol. 2024 Sep 25;12:1463047. doi: 10.3389/fbioe.2024.1463047 (PMC11461895; doi:10.3389/fbioe.2024.1463047)
Supplement: Supplementary file 2 [file DataSheet1.docx]

**Supplementary Figures**


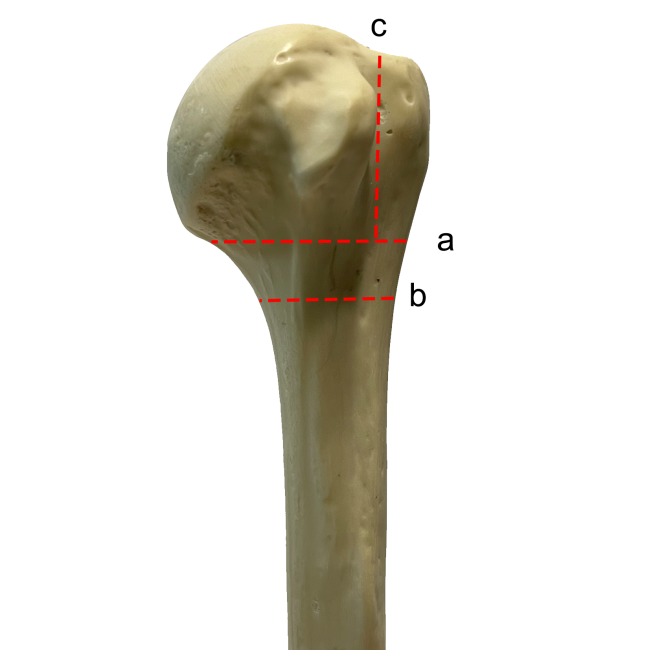


**Supplementary Figure 1.** Simulation of an AO 11-B3.2 fracture. The osteotomy lines are dotted red lines (the distance between lines a and b is 10mm).


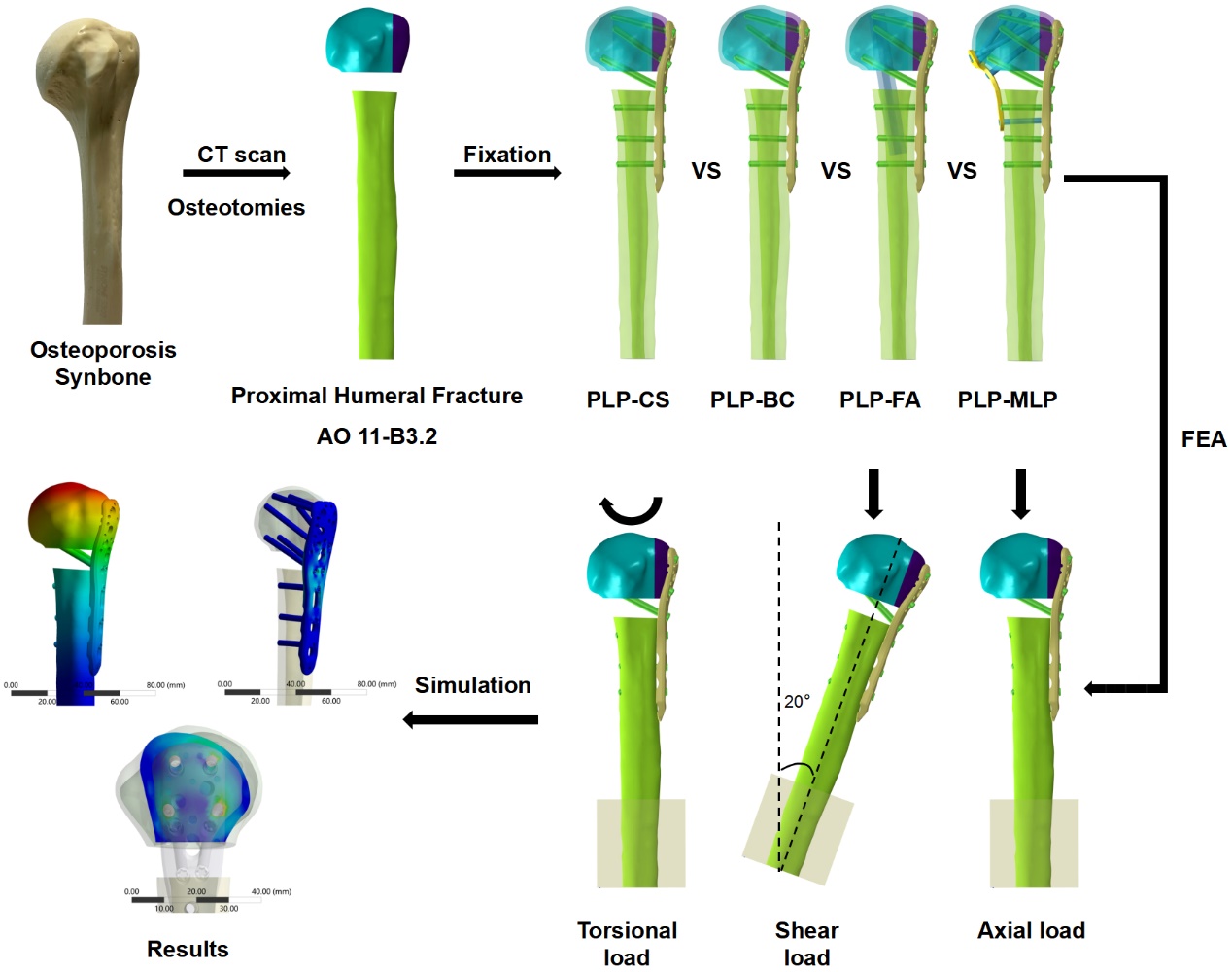


**Supplementary Figure 2.** The overall FEA workflow. PHF models fixed with different medial support methods were constructed using the CT data of the synthetic humerus, and load applications were applied according to the biomechanical experimental protocol to compare the biomechanical characteristics of each group.


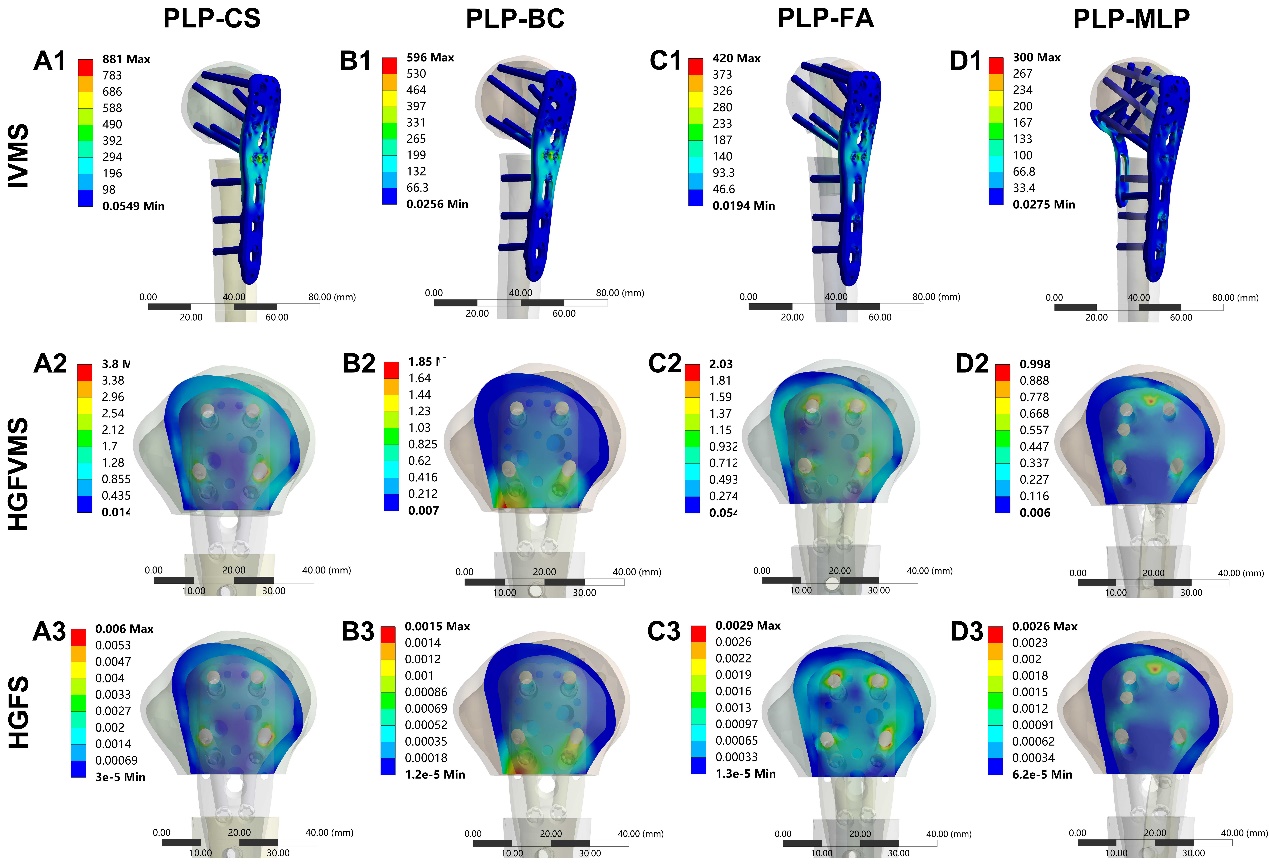


**Supplementary Figure 3.** Distribution of IVMS, HGFVMS, and HGFS for PHFs fixed by different medial support methods under shear loading. **A** PLP-CS; **B** PLP-BC; **C** PLP-FA; **D** PLP-MLP.


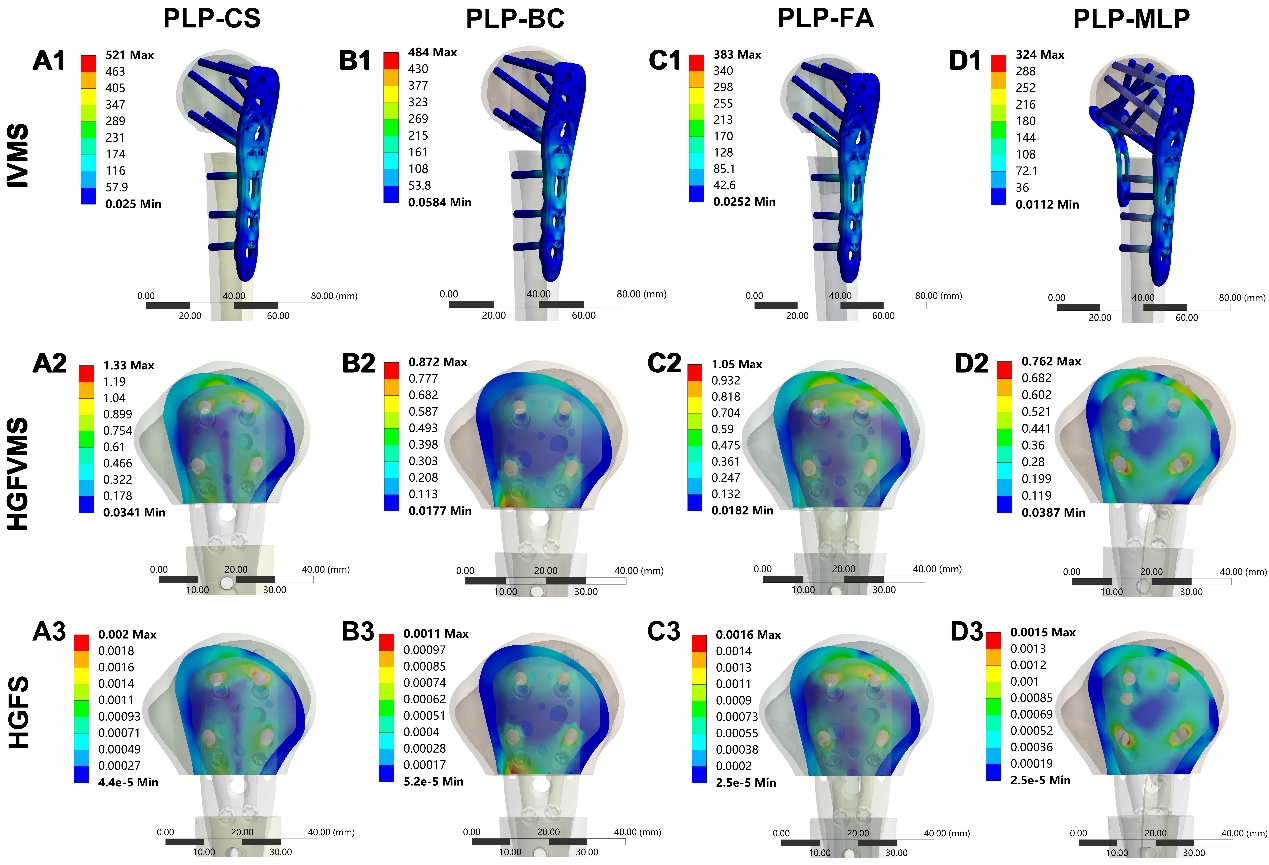


**Supplementary Figure 4.** Distribution of IVMS, HGFVMS, and HGFS for PHFs fixed by different medial support methods under torsional loading. **A** PLP-CS; **B** PLP-BC; **C** PLP-FA; **D** PLP-MLP.
